# Management practices regulate the response of Moso bamboo foliar stoichiometry to nitrogen deposition

**DOI:** 10.1038/srep24107

**Published:** 2016-04-07

**Authors:** Xinzhang Song, Honghao Gu, Meng Wang, Guomo Zhou, Quan Li

**Affiliations:** 1 The Nurturing Station for the State Key Laboratory of Subtropical Silviculture, Zhejiang A&F University, Lin’an, 311300, China; 2 Laboratory for Ecological Forecasting and Global Change, College of Forestry, Northwest Agriculture and Forest University, Yangling, 712100, China

## Abstract

Moso bamboo, well known for its high growth rate, is being subjected to increasing amounts of nitrogen deposition. However, how anthropogenic management practices regulate the effects of N deposition on Moso bamboo stoichiometry remains poorly understood. We observed the effects of two years of simulated N deposition (30, 60 and 90 kg N ha^−1^yr^−1^) on the foliar stoichiometry of Moso bamboo plantations under conventional management (CM) and intensive management (IM). Young bamboo had significantly greater foliar N and P concentrations and N:P ratios than mature plants (*P* < 0.05). IM significantly increased the foliar N concentrations of young bamboo and P concentrations of mature bamboo but decreased mature bamboo foliar N:P ratios (*P* < 0.05). Nitrogen increased foliar N and P concentrations in IM bamboo plantations, but the positive effects were diminished when the addition rate exceeded 60 kg N ha^−1^yr^−1^. Nitrogen increased foliar N concentrations but aggravated P deficiency in CM bamboo plantations. The positive effects of N deposition on foliar stoichiometry were influenced by management practices and bamboo growth stage. The effects of N deposition on foliar stoichiometry combined with anthropogenic management practices can influence ecosystem production, decomposition, and subsequent N and P cycles in Moso bamboo plantations.

The quantitative relationships between foliar nitrogen (N) and phosphorus (P) concentrations, as well as the effects of N and P limitation on plant growth under global climate change, are increasingly concerning in ecological stoichiometry theory^1^,^2^. N and P are essential for plant growth, and the foliar N:P ratio is thought to be useful for assessing N versus P limitation in primary production in terrestrial ecosystems^3^,^4^. Stoichiometric homeostasis varies with plant species, some are able to maintain their nutrient balance other not^5^,^6^. Foliar N:P stoichiometry can be strongly affected by environmental nutrient availability^7^,^8^,^9^ and plant growth stage^10^,^11^. For example, Wu *et al*.^10^ reported that the foliar N and P concentrations of three wetland plants were highest in the early stages of growth and the smallest in the middle growth stage.

The increasing consumption of fossil fuels and application of agricultural fertilizers have largely enhanced the amount of anthropogenic available N being input into ecosystems worldwide in the last few decades^12^,^13^, and the ecological effects are attracting attention. Previous studies have reported that N addition increased the foliar N concentration and N:P ratio, and both increases and decreases have been found in the subsequent P uptake capacity of plants^4^,^9^,^14^, including in fast-growing arbor species such as *Eucalyptus grandis*^15^ and hybrid poplar^16^.

Moso bamboo (*Phyllostachys pubescens* Mazel ex H. de Lehaie) is a monopodial giant bamboo widely distributed in southern China and neighboring countries, such as Japan and Korea^17^. As a highly representative plantation forest in the subtropical region of China, the Moso bamboo forest is currently the most important source of non-wood forest products in China^18^. The forest covers an area of 3.87 million ha, representing 70% of the Chinese bamboo forest area and 80% of the global distribution of Moso bamboo^18^,^19^. Due to the enormous ecological and socioeconomic benefits, Moso bamboo forests have expanded rapidly in subtropical China, with an annual increase of 3% in recent decades^18^.

Being greatly different from the arbor species, the woody rhizomatous plant Moso bamboo is well known for its startlingly high growth rate. New bamboo usually grows to a height of 10–20 m and a diameter of 8–16 cm at breast height within 35–40 days after germination from the soil in the spring. Afterwards, the height, diameter and volume remain unchanged because of the scarce secondary cambium, and the bamboo begins to slowly accumulate dry matter^20^,^21^,^22^. New bamboo begins to grow leaves in May of the current year during which it emerges. These initial leaves fall the next spring, and new leaves quickly reemerge. These re-growing leaves will continue to fall biennially until a life span of two years is reached^17^. Moreover, due to long-term management practices, Moso bamboo plantations have been characterized by years of alternating high- and low-recruitment of new bamboo^21^,^23^,^24^. Thus, the Moso bamboo plantations are unevenly aged forests with a two-year interval. How this growth pattern of Moso bamboo influences foliar stoichiometry is still unclear.

To maintain high productivity in Moso bamboo plantations and maximize the economic benefits, increasing numbers of farmers are adopting intensive management (IM) practices, such as the regular removal of understory vegetation, plowing, and fertilization^18^,^25^. Typically, conventional management (CM) consists of regularly harvesting bamboo trunks and shoots, without any other management practices^25^. IM practices have increased bamboo production but reduced soil microbial functional diversity^26^,^27^. Moreover, the IM practice has also accelerated soil organic carbon mineralization^28^,^29^, influenced N and P release from decomposing litter^30^, altered soil nutrient availability^31^, and thus affected bamboo nutrient balance. Our previous investigation showed that IM practices increased Moso bamboo foliar N and P concentrations^32^.

The average bulk deposition of N has increased from 13.2 kg N ha^−1^yr^−1^ in the 1980s to 21.1 kg N ha^−1^yr^−1^ in the 2000s across China^33^. Subtropical China, with a maximum annual N deposition rate of 63.53 kg N ha^−1^yr^−1^ 34, is predicted to become the region with the greatest N deposition in the world by 2030^35^. However, knowledge of the effects of N deposition on plant stoichiometry in forest ecosystems in China remains limited^36^,^37^. Most Moso bamboo plantations are distributed in the center of the region, with the greatest N deposition in China, both at the present time and in future predictions^30^. Thus, the effects of increasing N deposition on the foliar stoichiometry of managed Moso bamboo plantations would be affected by anthropogenic management practices. However, thus far, a lack of information about these interactions has limited our understanding of the combined effects of N deposition and anthropogenic management practices on the Moso bamboo forest ecosystem.

In this study, we investigated the variation of foliar N and P concentrations in Moso bamboo at different ages under IM and CM practices after exposure to simulated N deposition for two years. The objectives are to examine (1) how foliar N and P stoichiometry responds to management practices, (2) how foliar N and P stoichiometry responds to increasing N deposition, and (3) whether the effects of N deposition on foliar stoichiometry depend on management practices and growth stage.

## Results

### Foliar N and P concentrations

**Figure 1:**
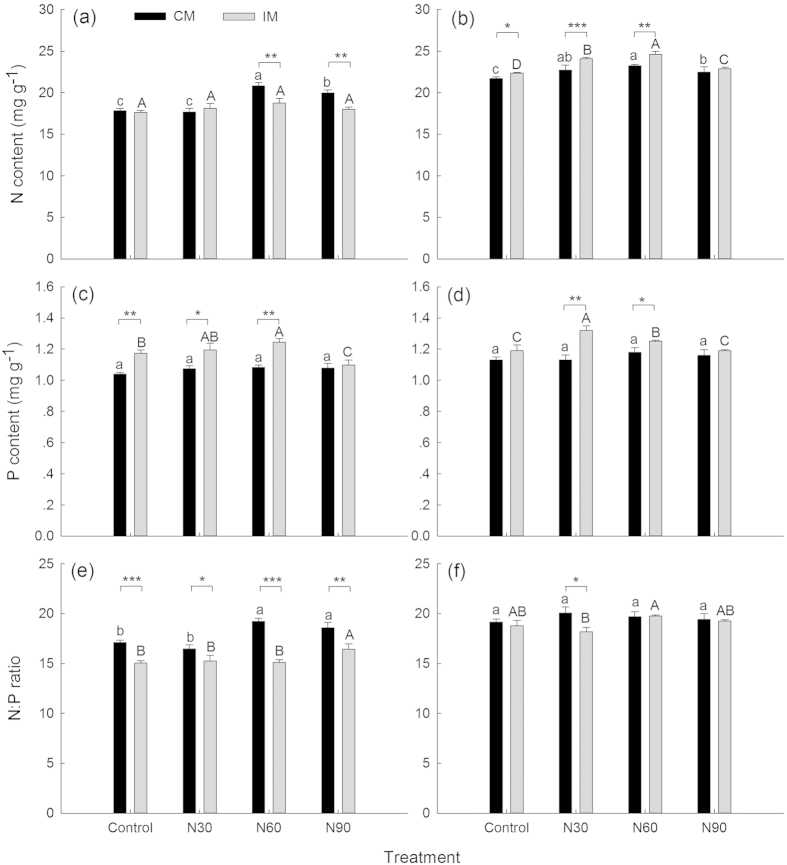
Mature Moso bamboo foliar N (**a**) and P (**c**) concentrations and the N:P ratio (**e**), and young Moso bamboo foliar N (**b**), and P (**d**) concentrations and the N:P ratio (**f**) under different management practices (CM: conventional management; IM: intensive management) and N treatments (control; N30: 30 kg N ha^−1^yr^−1^; N60: 60 kg N ha^−1^yr^−1^; N90: 90 kg N ha^−1^yr^−1^) (n = 3). Different lowercase letters indicate significant differences among N addition rates under CM treatments (*P* < 0.05). Different capital letters indicate significant differences among N addition rates under IM treatments (*P* < 0.05). Asterisks indicate significant differences between CM and IM at the same N addition rate (*P < 0.05, **P < 0.01, ***P < 0.001).

In CM plots, compared with the control, N addition significantly increased the foliar N concentration by 3.55–16.88% (Fig. 1a,b). However, these positive effects were diminished when the N addition rate was more than 60 kg N ha^−1^yr^−1^. Nitrogen addition did not significantly influence the foliar P concentration (Fig. 1c,d). In IM plots, compared with the control, N addition significantly increased the foliar N concentration of young bamboo by 2.33–10.25% (Fig. 1b) but did not affect that of mature bamboo (Fig. 1a). These positive effects on young bamboo foliar N were diminished when the N addition rate was more than 60 kg N ha^−1^yr^−1^. Nitrogen addition significantly increased the foliar P concentration by 5.04–10.92%, but excess N, such as 90 kg N ha^−1^yr^−1^, decreased the mature bamboo foliar P concentration by 6.48% (Fig. 1c,d).

Young bamboo foliar N concentrations were significantly larger upon IM than upon CM when the N addition level was less than 60 kg N ha^−1^yr^−1^, but mature bamboo foliar N concentrations showed a contrary trend under the N60 and N90 treatments, in which the foliar N concentrations were 9.93–9.96% lower in CM compared with IM plots (Fig. 1a,b). Mature bamboo foliar P concentrations were significantly greater upon IM than upon CM by 11.81–14.77% when the N addition rate was less than 60 kg N ha^−1^yr^−1^. Young bamboo showed a similar pattern under the N30 and N60 treatments (Fig. 1c,d). Young bamboo foliar N concentrations were always significantly greater than those of mature bamboo under all treatments (*P* < 0.01), and there was a general trend toward P concentrations being greater in young bamboo than in mature plants (Fig. 1, Table 1). Pearson correlation analysis showed that there was a significant correlation between N and P concentrations only in young bamboo (Table 2).

Three-way analysis of variance (ANOVA) showed that foliar N concentrations were significantly influenced by three factors—management type, N addition and growth stage—both individually and interactively, except for management type (Table 3). Similarly, foliar P concentrations were also significantly influenced by these three factors, both individually and interactively, except for the interaction between growth stage, management type and N addition.

### Foliar N:P ratios

In CM plots, compared with the control, N addition did not significantly influence young bamboo foliar N:P ratios (Fig. 1f), but it did significantly increase mature bamboo foliar N:P ratios by 8.64–12.38% in the N60 and N90 treatments (Fig. 1e). In IM plots, compared with the control, N addition did not significantly influence foliar N:P ratios except under N90 treatment for mature bamboo (Fig. 1e,f). Mature bamboo N:P ratios were always significantly greater in CM than in IM plots under all N addition levels (Fig. 1e), but this pattern was not observed for young bamboo except under N30 treatment (Fig. 1f). Young bamboo foliar N:P ratios were significantly larger than those for mature bamboo except in CM plots with N60 and N90 treatments (Table 1, Fig. 1e,f). Three-way ANOVA showed that the foliar N:P ratios were significantly influenced by the three factors both individually and interactively, except for the interaction between management type and N addition level (Table 3).

## Discussion

### Effects of management practices on foliar stoichiometry

Under N-free conditions, IM treatment significantly increased young bamboo foliar N (Fig. 1b) and mature bamboo foliar P (Fig. 1c), and it significantly decreased mature bamboo foliar N:P (Fig. 1e), being consistent with our previous investigation^32^. Widespread fertilization management is thought to result in an increase in the N:P ratios of the natural terrestrial ecosystem and a decline in soil N:P ratios in some cropland ecosystems because of greater solubility and loss of N^9^. In the present IM plots, owing to annual fertilization of 450 kg ha^−1^ (N:P_2_O_5_:K_2_O, 15:6:20, equating 67.5 kg N and 11.8 kg P ha^−1^yr^−1^), IM treatments significantly increased soil N and P contents and decreased the soil N:P ratio due to a greater increase in P content (+76.78%) than N (+9.35%) (Fig. S1), to some extent contributing to the changes in foliar stoichiometry.

In contrast to temperate ecosystems, tropical forests are generally considered N-rich but P-poor^38^. Han *et al*.^39^ also found that soil P was lower in subtropical areas than in temperate areas in China, which led to a similar pattern in foliar P, indicating a strong P limitation in subtropical China. In the present study, under N-free conditions, the significantly larger foliar P and smaller N:P ratios in both soil and mature bamboo, together with significantly larger foliar N in young bamboo upon IM treatments than upon CM, indicated that both N and P elements were deficient in the CM Moso bamboo forests. Our results corroborate the fact that current IM practices, especially fertilization, provide available N and P elements to Moso bamboo.

Plant N and P contents vary with the growth stage^1^,^10^,^40^. In the present study, the significantly greater foliar N and P in young bamboo than mature bamboo (Table 1, Fig. 1) indicated that young bamboo had a higher demand for N and P elements because after completing fast height and volume growth within two months, young bamboo begins a slow accumulation of dry matter^18^,^22^ and needs more N and P elements in its leaves to complete the primary production process. Moreover, when there is not sufficient N or P in CM plots, these elements are preferentially provided to young bamboo through underground rhizomes that connect young and mature bamboo and transfer nutrients among them^17^,^21^.

Mature bamboo had similar foliar N contents of 17.83 and 17.63 mg g^−1^ under CM and IM treatments without N addition, respectively (Fig. 1a), which are nearly identical to the levels reported by Elser *et al*.^41^ for 397 terrestrial plants (17.6 mg g^−1^), and slightly lower than those reported by Han *et al*.^39^ for 753 Chinese terrestrial plants (18.6 mg g^−1^) and by Reich and Oleksyn^42^ for 1251 world terrestrial plants (18.3 mg g^−1^). Foliar P concentrations of both young and mature bamboo under two management practices (1.04–1.19 mg g^−1^) were slightly lower than that of 753 Chinese terrestrial plants (1.21 mg g^−1^)^39^ but remarkably lower than those of 397 terrestrial plants (1.58 mg g^−1^)^41^ and 1251 world terrestrial plants (1.42 mg g^−1^)^42^. The growth rate hypothesis proposes that fast-growing organisms require relatively more P-rich RNA to support rapid rates of protein synthesis^1^. In the present study, as a fast-growing plant, Moso bamboo has a relatively low foliar P concentration compared to terrestrial plants across China^39^ and the world^41^,^42^, being inconsistent with the growth rate hypothesis, which may be due to the especially fast growth rate of Moso bamboo. Alternatively, the growth rate hypothesis alone may not dictate leaf stoichiometry^43^.

### Effects of N deposition on foliar stoichiometry and interactions with management practices and growth stage

Our results showed that N addition significantly increased the foliar N concentration (except for mature bamboo under IM treatment), increased the foliar P concentration under IM treatment (except at the highest rate of N addition (90 kg N ha^−1^yr^−1^)), and generally increased foliar N:P ratios of mature bamboo. Moreover, these effects depended on the management treatment and the bamboo growth stage (Table 3).

In CM plots, foliar N concentrations of both young and mature bamboo significantly increased with the N addition rate but began to decline when the N addition rate exceeded 60 kg N ha^−1^yr^−1^ (Fig. 1a,b), indicating that the N element is deficient in conventionally managed bamboo forests. In IM plots, however, only young bamboo foliar N significantly increased with the N addition rate, and it declined when the N addition rate exceeded 60 kg N ha^−1^yr^−1^ (Fig. 1a,b), indicating that the N element was still lacking for young Moso bamboo, even though fertilization (equating 67.5 kg N ha^−1^yr^−1^) significantly increased the soil N content under IM treatment (Fig. S1a). The positive effects of N addition on foliar N were also observed in previous studies^4^,^14^,^15^ and were consistent with our current findings. However, excessive N deposition (>60 kg N ha^−1^yr^−1^), in turn, could restrain N uptake. Aber *et al*.^44^ thought that the foliar N concentration would increase in the initial response to N input and then would decline upon excessive N deposition because of the limited availability of other resources essential for growth. In addition, excessive N input (high N addition combined with annual fertilization (67.5 kg N ha^−1^yr^−1^)) and atmospheric N deposition ((30.9 kg N ha^−1^yr^−1^)^45^ in IM plots) could decrease the activity of extracellular enzymes and thus decrease the net mineralization rate of soil N^46^.

Nitrogen addition can increase the P uptake capacity of plants by stimulating the activity of phosphatase in the rhizosphere^47^,^48^. In IM plots, foliar P concentrations significantly increased with the N addition rate (≤60 kg N ha^−1^yr^−1^) and were significantly greater than upon CM. However, in CM plots, this increase in foliar P concentrations with N addition did not happen (Fig. 1c,d) because P is deficient in the soil of CM plots, whereas annual fertilization (equating 11.8 kg P ha^−1^yr^−1^) provides abundant P to IM plots. The significantly lower soil P content in CM plots compared with IM plots supports this assumption (Fig. S1). The current result indicates that the extent to which N deposition affects Moso bamboo foliar P concentrations depends on the soil P condition, which is mainly controlled by fertilization in Moso bamboo plantations. In addition, our previous study on the effects of N deposition on Moso bamboo litter decomposition found that IM practices weakened the positive effects of N deposition on N loss but enhanced the positive effects on P loss in the early decomposition stage^30^. Moreover, IM practices decreased the diversity and biomass of understory plants^21^,^30^. These effects of IM practices together regulate the effects of N deposition on foliar stoichiometry.

In IM plots, the foliar P concentrations started to decline when the N addition rate exceeded 60 kg N ha^−1^yr^−1^ (Fig. 1c,d), indicating that moderate N deposition (≤60 kg N ha^−1^yr^−1^) can favor P uptake but excessive N deposition (> 60 kg N ha^−1^yr^−1^) will restrain P uptake. The soil acidification induced by high N may partly explain this result. The soil of our bamboo stands is acidic even in the CM plots (Fig. S1d) because of the severe N deposition in this region^45^. In the IM plots, high N addition (≥60 kg N ha^−1^yr^−1^), combined with annual fertilization (67.5 kg N ha^−1^yr^−1^) and atmospheric N deposition (30.9 kg N ha^−1^yr^−1^)^45^, can largely increase the solution loss of NO_3_^-^ and cations from soil and increase H^+^ content in soil through the reaction of NH_4_^+^ nitrification^49^,^50^, resulting in a significant decline of soil pH compared with low N addition (≤30 kg N ha^−1^yr^−1^) and CM plots (Fig. S1d). The decline in soil pH can decrease the mineralization rates of soil P and available P^49^,^51^, and it can subsequently lead to a decline in foliar P. In addition, excessive N deposition can increase the dissolution of aluminum ions, which can also inhibit P uptake^49^,^52^. Previous studies also demonstrated that high N deposition decreased foliar P concentrations of *Abies balsamea* and *Betula papyrifera* var. *cordifolia* in subalpine forests of the northeastern United States^53^. Wessman *et al*.^54^ suggested that foliar chemistry (such as N and P concentrations) responses to N deposition could be regarded as predictors of the earliest stages of N saturation. Our results showing that N and P concentrations began to decline when the N addition rate was more than 60 kg N ha^−1^yr^−1^ imply that the N deposition level of 60 kg N ha^−1^yr^−1^ might be presumed as a threshold for positive/negative ecological effects on Moso bamboo growth or as a marker for whether Moso bamboo forests have reached N saturation.

Nitrogen deposition can increase the N:P ratio of plants and soils in terrestrial ecosystems^9^,^55^. In the present study, N addition significantly influenced soil N and P contents and N:P ratios (Fig. S1). However, our Pearson correlation analysis showed that soil N and P contents and N:P ratios did not have significant correlations with foliar N and P concentrations or N:P ratios (*P* > 0.05, data not shown), except for soil N and foliar N under IM treatment (*P* = 0.025, data not shown). This result likely occurred because the foliar stoichiometry of Moso bamboo is controlled by multiple environmental factors aside from soil nutrients. Mature bamboo foliar N:P ratios remained stable at a value of 15 in IM plots under moderate N addition (≤60 kg N ha^−1^yr^−1^) and were always significantly lower than that in CM plots regardless of the N addition rate (Fig. 1e), supporting the assumption that there is a positive relationship between high N input and high N:P ratios. This relationship is strengthened by P limitation^7^ and also suggests that increasing N deposition would aggravate P limitation in CM Moso bamboo forests. These results indicate that the effects of N addition on the mature bamboo foliar N:P ratio are regulated by the effects of management practices.

Young bamboo foliar N:P ratios in the range of 18 to 20 did not show significant variation among N addition rates or management treatments (Fig. 1f), indicating that young bamboo had an intrinsically higher foliar N:P ratio than mature bamboo. Young bamboo contains higher foliar N and P concentrations than mature bamboo (Fig. 1) for more dry matter accumulation^21^,^22^. Moreover, the demand for N is greater than for P, which may result in a larger stable N:P ratio in young bamboo than in mature bamboo. Our findings indicate that the stable foliar N:P ratio may change with the Moso bamboo growth stage, being low for mature bamboo and high for young bamboo, and the effects of N addition on the foliar N:P ratio also vary with the Moso bamboo growth stage. Three-way ANOVA confirmed this conclusion.

Increasing N deposition can act together with increasing atmospheric CO_2_ to improve the growth and productivity of forests and thus enhance carbon sequestration capacity^56^, which is favorable to IM Moso bamboo forests. In CM Moso bamboo forests, however, P limitations would significantly constrain the N deposition fertilization effects that improve C storage in biomass by promoting growth^9^. Therefore, it is recommended to apply fertilizer, especially phosphate fertilizer, to CM Moso bamboo forests.

## Conclusion

Nitrogen deposition generally increased the foliar N and P concentrations and N:P ratios of intensively managed Moso bamboo but aggravated the P deficiency of conventionally managed Moso bamboo. When N deposition exceeded 60 kg N ha^−1^yr^−1^, these positive effects were diminished but negative effects could occur. Moreover, these positive effects were regulated by management practices and depended on the Moso bamboo growth stage. Findings from this study suggested that the combination of anthropogenic management practices should be taken into account when estimating the ecological consequences of increasing N deposition.

## Materials and Methods

### Study site

The study site was located in Qingshan Town, Lin’an City (30°14′N, 119°42′E), Zhejiang Province, China. The area has a monsoonal subtropical climate with four distinct seasons. The mean annual precipitation is 1,420 mm, and the mean annual temperature is 15.6 °C, ranging from 24 °C in July to 3 °C in January. The area receives an average of approximately 1,847 hours of sunshine per year and features an average of 230 frost-free days per year.

The conventionally managed Moso bamboo plantations were originally established in the late 1970s from native evergreen broadleaf forest in sites with similar topography (southwest slope of approximately 6 degrees) and soil type (Ferrisols derived from granite)^30^. The IM practices, including annual fertilization, plowing, and weeding with herbicide, have been conducted since 2001. In September of each year, compound fertilizer (N:P_2_O_5_:K_2_O, 15:6:20, 450 kg ha^−1^) is manually and evenly scattered on the soil surface and followed with a deep plowing to 0.3 m, which is equivalent to an annual addition of 67.5 kg N, 11.8 kg P, and 74.7 kg K per hectare. CM practices only selectively and regularly harvest bamboo trunks and shoots, which is also done in IM. Except for harvesting, conventionally managed bamboo forests did not receive any other management practices^30^.

### Experimental design and sampling

In November 2012, 24 plots of 20 × 20 m were established. The initial stand and soil characteristics of both the 12 CM plots and 12 IM plots are summarized in Song *et al*.^30^. Each plot was surrounded by a 20 m-wide buffer zone to avoid disturbing nearby plots. According to the local N deposition rate of 30.9 kg N ha^−1^yr^−1^ 45 and the widely used method (double and triple the local N deposition rate) in previous studies simulating N deposition^57^,^58^, three N addition treatments, low-N (N30, 30 kg N ha^−1^yr^−1^), medium-N (N60, 60 kg N ha^−1^yr^−1^), high-N (N90, 90 kg N ha^−1^yr^−1^), and a control (no added N) with three replicates were conducted randomly in each Moso bamboo plantation management type. Starting in January 2013, quantified NH_4_NO_3_ was weighed according to the N addition rate, mixed with 10 L of water and sprayed evenly onto the forest floor of each plot using an electric sprayer at the beginning of every month for a total of 12 equal applications over the entire year throughout the experiment period. These water-based additions amounted to an increase of 0.3 mm of rainfall per year. Each control plot received 10 L of N-free water.

### Sample collection

In the local region, bamboo trunks are usually harvested after growing for 4 years. The present Moso bamboo stands only include two growth stages: young bamboo growing out in the spring of 2014 (one-year-old) and mature bamboo growing out in the spring of 2012 (three-year-old). Therefore, the two-year-old leaves (growing out during the spring of 2013 from mature bamboo emerging in 2012) and one-year-old leaves (growing out during the spring of 2014 from young bamboo emerging in 2014) all fell in the spring of 2015.

After two years of N addition, sampling was performed in January 2015 to examine the short-term responses of foliar stoichiometry to N deposition. The diameter at breast height (DBH) of each bamboo plant in each plot was measured, and the mean DBHs of young and mature bamboo were calculated in each plot. Five young and five mature bamboo plants with the DBH closest to the mean were selected from each plot, and the leaves in the middle and upper parts of the canopy were sampled and mixed according to the corresponding bamboo age. The leaf samples were collected and transported to the laboratory in insulated cases at 4 °C and then were oven-dried at 105 °C for 30 minutes and 65 °C to a constant weight. Oven-dried leaves were milled for analysis of N and P content.

At the same time, to better explain the change in foliar stoichiometry under different treatments, the top 20 cm portions of the soils were also collected randomly from five spots in each plot with a soil auger (3.5 cm diameter) and then mixed together. The soil samples were air-dried for a few months in the laboratory for chemical analysis (Fig. S2). Soil pH was measured using a pH meter (FE20, Mettler Toledo, Switzerland) after shaking the soil water (1:2.5 w/v) suspension for 30 min. The total N concentrations of both plant and soil samples were determined using a CN automatic analyzer (Sumigraph NC-80, Shimadzu, Japan). The P concentrations of both plant and soil samples were determined using a modified Kjeldahl method followed by colorimetric analysis^59^.

### Data and statistical analysis

One-way ANOVA and least significant difference (LSD) multiple comparisons were used to determine the significance of differences between foliar N and P concentrations and mass-based N:P ratios among the four simulated N treatments in each plantation management type and growth stage, or between two plantation management types in each N treatment and growth stage, or among growth stages in each management type and N treatment. Similar statistical analyses were also used on soil N and P contents and N:P ratios among the four simulated N addition treatments in each plantation management type as well as between two plantation management types in each N treatment. The results showed that the assumptions of homogeneity of variance were met. A three-way ANOVA method was used to test the effect of the interaction of three factors (i.e., N addition, management practices and growth stage) on foliar stoichiometry. Pearson correlation analysis was performed to test the correlation of foliar N and P concentrations between young and mature bamboo, and of soil and leaf stoichiometry. All analyses were conducted using SPSS (Statistical Package for the Social Sciences) 16.0 for Windows (SPSS Inc., Chicago, Illinois).

## Additional Information

**How to cite this article**: Song, X. *et al*. Management practices regulate the response of Moso bamboo foliar stoichiometry to nitrogen deposition. *Sci. Rep*. **6**, 24107; doi: 10.1038/srep24107 (2016).

## Supplementary Material

Supplementary Information

## Figures and Tables

**Table 1:** The significance level of effects of Moso bamboo growth stage on foliar stoichiometry under different treatments (n = 3, df = 1).

Treatment	N		P		N:P	
	F	Sig.	F	Sig.	F	Sig.
CM						
Control	343.6	***	48.6	**	80	**
N30	410.6	***	7.4	ns	81.1	**
N60	118.7	***	24.7	**	2.1	ns
N90	32.9	**	9.3	*	3.5	ns
IM						
Control	6225.2	***	0.481	ns	115.5	***
N30	392.1	***	17.7	*	49.2	**
N60	240.8	***	0.05	ns	716.8	***
N90	523.8	***	22.8	**	76.9	**

CM: conventional management; IM: intensive management; N: nitrogen; P: phosphorus. Asterisks indicate that the young bamboo foliar N and P concentrations and N:P ratios are significantly larger than those of mature bamboo (*P < 0.05, **P < 0.01, ***P < 0.001). ns, no significance.

**Table 2:** Pearson correlation coefficients between the foliar nitrogen (N) and phosphorus (P) concentrations of Moso bamboo at different growth stages under conventional management (CM) and intensive management (IM) (n = 12).

Growth stage	CM	IM
Mature	0.41	0.46
Young	0.60*	0.65*

^*^The correlation is significant at the α = 0.05 level.

**Table 3:** The significance levels in three-way ANOVA for the effects of management type, N level, bamboo age and their interactions on foliar nitrogen (N), phosphorus (P) and N:P ratios of Moso bamboo.

Source of variation	N			P			N:P		
	df	F	Sig.	df	F	Sig.	df	F	Sig.
Management type	1	0.0	0.9999	1	155.1	0.0001	1	151.1	0.0001
Bamboo age	1	1889.3	0.0001	1	83.5	0.0001	1	468.9	0.0001
N addition level	3	65.6	0.0001	3	15.5	0.0001	3	19.5	0.0001
Management × Age	1	87.9	0.0001	1	2.1	0.1587	1	54.3	0.0001
Management × N level	3	13.1	0.0001	3	12.4	0.0001	3	2.6	0.0656
Age × N level	3	14.6	0.0001	3	2.2	0.1122	3	6.3	0.0017
Management × Age × N level	3	9.7	0.0001	3	5.8	0.0027	3	16.8	0.0001
