# Supplementary material for: Management practices regulate the response of Moso bamboo foliar stoichiometry to nitrogen deposition
Source: Sci Rep. 2016 Apr 7;6:24107. doi: 10.1038/srep24107 (PMC4823648; doi:10.1038/srep24107)
Supplement: Supplementary Information [file srep24107-s1.pdf]

Management practices regulate the response of Moso bamboo foliar stoichiometry to nitrogen deposition

Xinzhang Song[1*], Honghao Gu[1], Meng Wang[2], Guomo Zhou[1], Quan Li[1]

[1]*The Nurturing Station for the State Key Laboratory of Subtropical Silviculture, Zhejiang A&F University, Lin'an, 311300, China*

[2]*Laboratory for Ecological Forecasting and Global Change, College of Forestry, Northwest Agriculture and Forest University, Yangling, 712100, China*

[*]Corresponding author: Tel: (86) 571-63741816; Fax: (86) 571-63742899

E-mail address: songxinzhang@gmail.com;

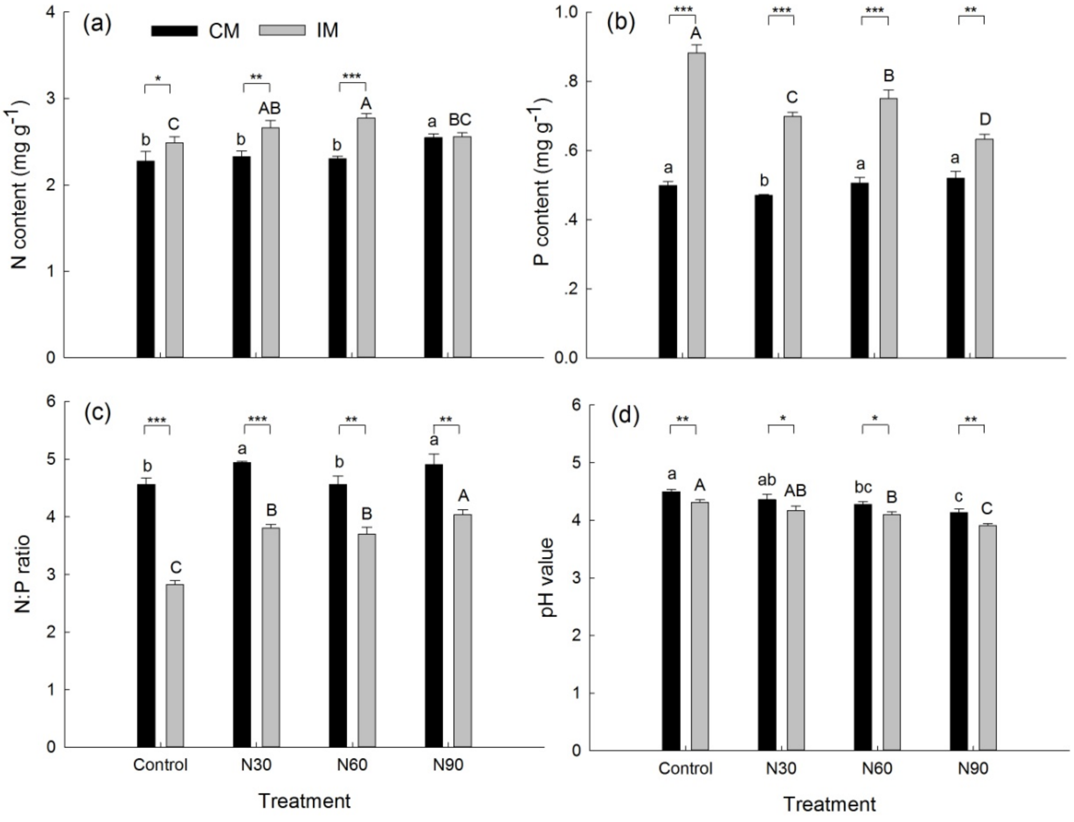

Figure S1. Moso bamboo forests soil N (a), P (b) concentrations, N:P ratios and pH values under different management practices (CM: conventional management; IM: intensive management) and N addition treatments (the control; N30: 30 kg N $ha^{-1}yr^{-1}$; N60: 60 kg N $ha^{-1}yr^{-1}$; N90: 90 kg N $ha^{-1}yr^{-1}$) (n=3). Different lowercase letters indicate significant differences among N addition rates under CM treatments (P<0.05). Different capital letters indicate significant differences among N addition rates under IM treatments (P<0.05). Asterisks indicate significant differences between CM and IM at the same N addition rate (*P < 0.05, **P < 0.01, ***P < 0.001).
